# Thyrotoxicosis in MCT8 deficiency

**DOI:** 10.1210/clinem/dgaf707

**Published:** 2026-01-09

**Authors:** Kristien Boelaert, Andrew Bauer, Anne R Cappola, Krishna Chatterjee, Johannes W Dietrich, Lindsey Nicol, Luca Persani, Salman Razvi, Athanasia Stoupa

**Affiliations:** Department of Applied Health, School of Health Sciences, College of Medicine and Health, University of Birmingham, Birmingham B15 2FG, UK; The Thyroid Center, Division of Endocrinology and Diabetes, The Children's Hospital of Philadelphia, Department of Pediatrics, Perelman School of Medicine, University of Pennsylvania, Philadelphia, PA 19104, USA; Division of Endocrinology, Diabetes, and Metabolism, Perelman School of Medicine, University of Pennsylvania, Philadelphia, PA 19104, USA; Institute of Metabolic Science, University of Cambridge, Cambridge CB2 0QQ, UK; Diabetes, Endocrinology and Metabolism Section, Department of Internal Medicine I, St. Josef Hospital, Ruhr University Bochum, Bochum, NRW D-44791, Germany; Diabetes Centre Bochum/Hattingen, St. Elisabeth-Hospital Blankenstein, Hattingen, NRW D-45527, Germany; Centre for Diabetes Technology, Catholic Hospitals Bochum, Ruhr University Bochum, Bochum, NRW D-44791, Germany; Centre for Rare Endocrine Diseases, Ruhr Centre for Rare Diseases (CeSER), Ruhr University Bochum and Witten/Herdecke University, Bochum, NRW D-44791, Germany; Centre for Thyroid Medicine KKB, Catholic Hospitals Bochum, Ruhr University Bochum, Bochum, NRW D-44791, Germany; Division of Pediatric Endocrinology and Diabetes, Oregon Health & Science University, Portland, OR 97239, USA; Department of Medical Biotechnology and Translational Medicine, University of Milan, Milan 20100, Italy; Department of Endocrine and Metabolic Diseases, IRCCS Istituto Auxologico Italiano, Milan 20145, Italy; Translational and Clinical Research Institute, Newcastle University, Newcastle upon Tyne NE1 3BZ, UK; Paris Regional Screening Program Department, Centre Régional de Dépistage Néonatal (CRDN), Ile de France, 75015 Paris, France; Pediatric Endocrinology, Diabetology and Gynecology Department, Necker Children's University Hospital, Assistance Publique Hôpitaux de Paris-APHP, Cochin Institute, U1016 and IMAGINE Institute Affiliate, Paris Cité University, 75015 Paris, France

**Keywords:** thyrotoxicosis, MCT8 deficiency, Allan–Herndon–Dudley syndrome, MCT8, thyroid hormone, *SLC16A2*, thyroid dysfunction

## Abstract

Monocarboxylate transporter 8 (MCT8) deficiency, also known as Allan–Herndon–Dudley syndrome, is a rare, severely debilitating, and life-limiting genetic disorder caused by variants in the *SLC16A2* gene that render the MCT8 thyroid hormone transporter partially or completely dysfunctional. MCT8 is highly expressed throughout the body, including the brain. Its deficiency disrupts thyroid hormone homeostasis and is associated with 2 distinct concomitant clinical presentations: persistent peripheral thyrotoxicosis resulting from elevated serum levels of triiodothyronine and neurodevelopmental impairment arising from low thyroid hormone levels in the brain. The disorder severely impacts quality of life and reduces life expectancy to a median of 35 years due to a range of clinical sequelae, with approximately 30% of affected individuals dying during childhood. Recognition and treatment of thyrotoxicosis are crucial to prevent associated symptoms and long-term sequelae.

## Thyroid physiology

**Figure 1.**
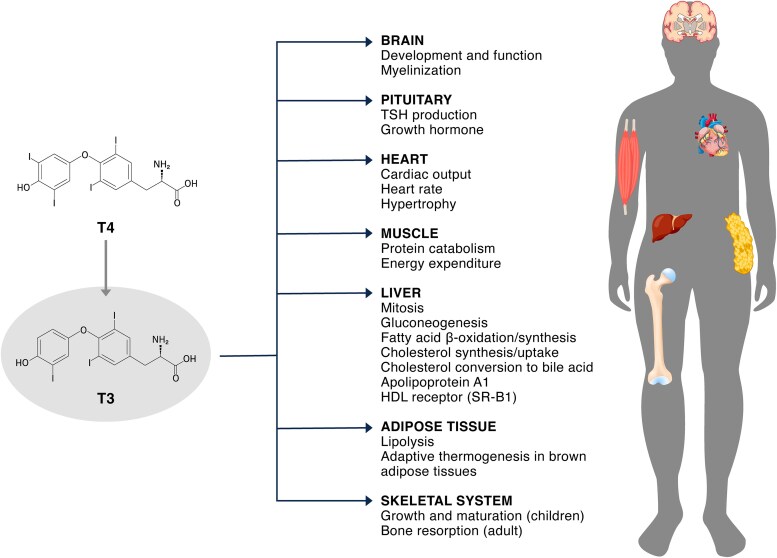
The effects of triiodothyronine (T3) on physiological functions. (2). Abbreviations: HDL, high-density lipoprotein; SR-B1, scavenger receptor, class B type 1; T4, thyroxine; TSH, thyroid-stimulating hormone.

Thyroid hormones (THs) play an essential role in the development and function of nearly all tissues in the human body (1-5). TH signaling is crucial for normal neurodevelopment and is a key regulator of metabolic processes in various tissues, as shown in Fig. 1 (2).

The effects of TH are broadly elicited through the binding of bioactive triiodothyronine (T3) to nuclear thyroid receptors (TRs), TRα and TRβ. The predominant form of TH released from the thyroid is thyroxine (T4), which is converted within cells into either T3 or the inactive metabolite reverse T3 (rT3) by deiodinase enzymes (DIO1-3); the conversion of T4 to T3 occurs mainly through DIO2, with a smaller contribution from DIO1, while DIO3 mediates T4 deactivation to rT3 (1, 5, 6). Thyroid signaling is a tightly regulated physiological process mediated by the hypothalamus-pituitary-thyroid axis (4).

## What is MCT8 deficiency?

Monocarboxylate transporter 8 (MCT8) deficiency, also known as Allan–Herndon–Dudley syndrome (AHDS), is an X-linked disorder that primarily affects male individuals, although a few confirmed cases have been reported in female individuals (7). Prevalence estimates range from fewer than 1 in 1 000 000 to 1 in 70 000 males (8, 9). Although rare, the disorder has a profound impact on the lives of these patients and their caregivers, including a requirement for lifelong care, an increased psychosocial burden for caregivers, and reduced life expectancy (9-11).

TH transporter proteins facilitate the transport of T3 and T4 across the cell membrane (1, 6). MCT8 is a critical, highly specific TH transporter expressed throughout the body and is encoded by the solute carrier family 16 member 2 gene (*SLC16A2*) located at chromosome Xq13.2 (3, 4, 12, 13). The *SLC16A2* gene spans 6 exons, 5 introns, and approximately 110 kb of the X chromosome (1, 14, 15). The MCT8 protein is composed of 539 amino acids with 12 predicted transmembrane domains, structural features that can influence the functional impact of variants (14, 16). A spectrum of pathogenic variants in *SLC16A2* has been identified, which can impact MCT8 transporter function, and the effects of these variants range from causing partial to complete protein loss of function (4, 17). Reported variants in *SLC16A2* are generally maternally inherited but can develop spontaneously in utero (4, 13, 18-23). These pathogenic variants, including missense, nonsense, frameshift, splice variants, and larger deletions/duplications in *SLC16A2,* lead to a broad range of phenotypes (1, 3, 4). Genotype–phenotype correlations have shown that the loss of function impact of variants is correlated with disease severity, and variant pathogenicity status related to Human Phenotype Ontology–defined symptomatology (20, 21).

Transport of TH across the blood-brain barrier and into neuronal tissues is MCT8-dependent. In patients with MCT8 deficiency, anomalies in neuronal differentiation, myelination, and synaptogenesis occur during intra-uterine brain development, caused by reduced levels of T3 and T4 in the central nervous system (3, 4, 24). Loss of MCT8 function is thought to alter the sensitivity of the hypothalamus and pituitary to TH and increase T4 “trapping” in the kidneys, resulting in elevated serum T3 levels (4). In addition, based on in silico analysis, reduced MCT8 activity may lead to impaired transport of T4 out of the thyroid, resulting in an accumulation of intrathyroidal T4 and increased conversion to T3 (5, 25). In the periphery, TH enters cells via alternative transporters such as the highly homologous MCT10 (18, 26).

Thus, 2 distinct concomitant clinical presentations result from the opposing thyroid dysfunction in the brain and periphery. Neurological symptoms arise from central hypothyroidism due to reduced T3 in the brain, resulting in hypotonia of the head and axis, dystonia in the limbs, and often profound deficits in neurocognitive and neuromuscular development. Symptoms in other organs and body systems, such as failure to thrive, muscle wasting, tachycardia and arrhythmia, hypertension, and sweating, occur secondary to elevated circulating T3 levels (7, 10, 18, 19, 24, 27).

## Thyrotoxicosis

Thyrotoxicosis is characterized by elevated tissue TH levels throughout the body, most commonly caused by Graves disease, toxic multinodular goiter, and toxic adenoma, and may present as either overt (low serum thyrotropin [thyroid-stimulating hormone; TSH] with high T3 and/or T4) or subclinical (low serum TSH with normal T3 and T4 concentrations) (28). A typical presentation of thyrotoxicosis can include weight loss, osteoporosis, neuropsychiatric symptoms, and cardiovascular complications such as atrial fibrillation (19, 27, 29, 30). The relationship between TH levels and outcome is complex, with excess T3 predictive of all-cause and cardiovascular mortality (31-33).

Thyrotoxicosis in MCT8 deficiency presents a complex clinical scenario and is associated with challenges in diagnosis, management, and treatment. Diagnosis is impeded by the rarity of MCT8 deficiency and nonspecific symptoms upon presentation. The median age at diagnosis is estimated to range from 18 to 29 months, with recent evidence suggesting that the delay between symptom onset and genetic confirmation of MCT8 deficiency has shortened in recent years, likely due to increased disease awareness and wider access to next-generation sequencing technologies (10, 19, 22). Standard TH testing for congenital thyroid disorders does not detect MCT8 deficiency, given that serum T3 levels do not increase until 4 to 6 months of age, and standard tests typically include TSH with reflexive T4, and not T3 or rT3 (9). Although serum T3 levels are variable in the first few days of life, neonatal concentrations of inactive rT3 are low and could be detected through newborn screening, with rT3 levels or T3/rT3 ratio, as determined by liquid chromatography–tandem mass spectrometry (LC-MS/MS), used to diagnose MCT8 deficiency from birth (34). Understanding the relationship between MCT8 deficiency and thyrotoxicosis is critical for clinicians to effectively manage the symptoms of this disorder, both acutely and over the patient's lifespan. By delineating this complex relationship, we aim to improve the recognition and management of MCT8 deficiency-induced thyrotoxicosis, which may ultimately enhance therapeutic targets and patient outcomes.

## Methods

In May 2025, we conducted a review of the literature incorporating keyword searches of PubMed using terms (alone or combinations thereof) that included (or were related to): *AHDS*, *Allan–Herndon–Dudley syndrome*, *MCT8 deficiency*, *MCT8*, *SLC16A2*, *thyroid hormone*, *thyrotoxicosis*, *quality of life*, *cardiovascular*, *sleep*, *weight*, *hepatic*, and *bone*. Searches were limited to English-language articles. Relevant studies were identified, tabulated, and assessed for inclusion, and reference lists of selected articles were screened for additional sources.

## Results

### Mechanisms underlying thyrotoxicosis in MCT8 deficiency

**Figure 2.**
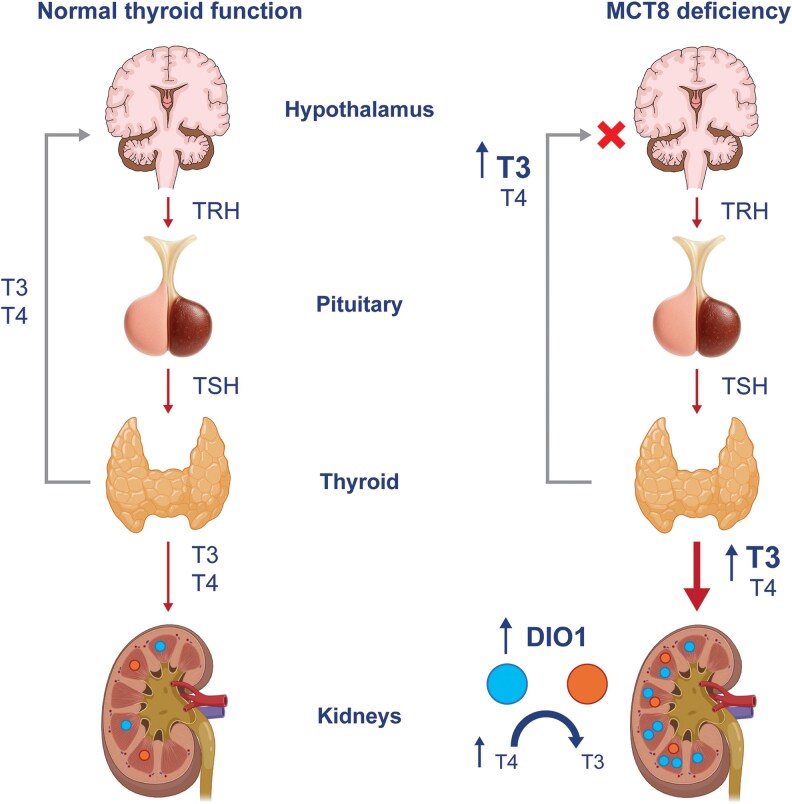
Proposed mechanisms responsible for the characteristic thyroid hormone (TH) profile in patients with monocarboxylate transporter 8 (MCT8) deficiency (4, 5, 25, 36, 37). In the brain, MCT8 is expressed in human tanycytes, thyrotropin-releasing hormone (TRH)-expressing neurons, and folliculostellate cells of the pituitary, and it is likely a key regulator of TH homeostasis in the hypothalamic-pituitary-thyroid axis. Loss of MCT8 function results in high circulating triiodothyronine (T3) levels that suppress TRH secretion from the hypothalamus and thyroid-stimulating hormone (TSH) secretion from the pituitary. This is accompanied by reduced secretion of thyroxine (T4) from the thyroid, due to both decreased TSH secretion from the pituitary, the direct effect of MCT8 deficiency in the thyrocyte membranes, impairing TH efflux, as well as the increased peripheral conversion of T4 to T3. This leads to an increase in intrathyroidal T4 and T3 and perturbation of the feedback mechanism. In the kidneys, increased activity of the type I iodothyronine deiodinase (DIO1) enzyme responsible for T4 to T3 conversion is accompanied by decreased excretion and “trapping” of T4. This is thought to result in rapid conversion of trapped T4 to T3 and the release of T3 into the circulation by alternative transporters.

Patients with MCT8 deficiency typically present with a characteristic profile of serum TSH within the normal age-specific range, low serum T4, elevated serum T3, and low serum rT3. Symptoms of thyrotoxicosis are likely a result of elevated free serum T3 and its uptake into peripheral tissues through alternative transporters (19, 35). The pathophysiology of this distinct TH fingerprint is yet to be fully elucidated, but it is thought to result from multiple processes, as shown in Fig. 2 (4).

These studies, largely based on murine models that do not fully replicate human physiology, highlight the need for further investigation into the precise mechanisms of TH dysregulation in MCT8-deficient patients.

### Manifestations of elevated serum T3 in patients with MCT8 deficiency

The effects of thyrotoxicosis in patients with MCT8 deficiency affect several organ systems. The key clinical presentations of thyrotoxicosis in this patient group are disturbed sleep, weight loss or lack of weight gain, muscle wasting, general restlessness, recurrent infections, and cardiac abnormalities (tachycardia, arrhythmia, systolic hypertension, increased risk of atrial fibrillation). Frequent fractures due to osteoporosis have also been noted. Regular infections and bone fractures occur secondary to poor nutrition, with decreased weight bearing compounding the risk for fractures (19, 38-44).

The first manifestation of thyrotoxicosis in individuals with MCT8 deficiency is often failure to thrive within the first year (19). Pregnancy and the first few months of life are typically uneventful, with nonspecific symptoms of global developmental delay and hypotonia arising at around 2 to 3 months. One natural history study showed a median age of symptom onset of 4 months (19). These nonspecific symptoms are typically the reason for the first presentation to a healthcare professional (HCP). In this setting, failure to thrive is often attributed to reduced caloric intake and feeding difficulties due to co-existing hypotonia and lack of head control, until it becomes clear that there is also an important metabolic component. Early recognition and intervention are critical to mitigating the long-term consequences of thyrotoxicosis on patient outcomes and quality of life (12).

### Metabolic dysregulation

Thyrotoxicosis induces a hypermetabolic state, characterized by an elevated basal metabolic rate and increased physiological activity, leading to weight loss, increased energy expenditure, and heat production (45). T3 is a key regulator of body weight. At the cellular level, elevated serum T3 levels exert pleiotropic effects on multiple metabolic pathways, enhancing the activity of enzymes involved in carbohydrate, fat, and protein metabolism, increasing (45, 46):

- Gluconeogenesis and glycogenolysis in the liver
- Lipolysis in adipose tissues
- Proteolysis and muscle catabolism, contributing to muscle wasting and weakness

TH-mediated upregulation of mitochondrial biogenesis and oxidative phosphorylation further adds to cellular energy and heat production (47).

Individuals with MCT8 deficiency are usually born with weight and head circumference within the normal range. However, at the time of first presentation to a HCP, patients typically exhibit a lower weight percentile with noticeable muscle weakness. Failure to gain weight is an important prognostic factor for patients with MCT8 deficiency. One study showed that individuals who did not achieve normal body weight between the ages of 1 and 3 years had an increased risk of death compared with patients who had a normal body weight for age (3, 4, 19).

Studies of adults diagnosed with hyperthyroidism have shown that their muscle mass is reduced by approximately 20% and muscle strength is reduced by 40% (48). Many patients with MCT8 deficiency have low muscle mass, likely directly related to elevated serum T3 (19). Improvement in weight and muscle tone is often recognized by parents and carers as a factor that would aid in improving the quality of life of these patients (49).

### Cardiovascular effects

Functional actions of TH in the cardiovascular system include the modulation of heart rate and rhythm, blood pressure, output, and stroke volume. T3 modulates the expression of important cardiac genes, both positively and negatively. Nongenomic effects of T3 in the heart typically occur at the plasma membrane and regulate ion transporter activity. Free serum T3 is positively correlated with heart rate, including in euthyroid individuals, and directly mediates increased cardiac contractility (50-53).

Symptomatic consequences and long-term sequelae of thyrotoxicosis are frequently seen in the heart. These include thyrotoxic cardiomyopathy (which may progress to heart failure) and electrocardiogram (ECG) changes, including tachycardia and tachyarrhythmias, altered QTc interval, ST segment elevation, T-wave inversion, and other irregularities (54, 55). Untreated thyrotoxicosis is associated with an increased risk of morbidity and mortality and has been associated with an increased risk of major cardiovascular events (29, 56, 57). Even mild forms of thyrotoxicosis may be associated with increased cardiovascular mortality (58). Manifestations of cardiac hyperthyroidism frequently include symptoms such as palpitations, hyperdynamic precordium, exercise intolerance, exertional dyspnea, anginal chest pain, and peripheral edema (50). Upon investigation, patients may have systolic hypertension, cardiac hypertrophy, atrial fibrillation, and, in severe cases, heart failure (50). Approximately 1% of all patients with thyrotoxicosis without MCT8 deficiency develop thyrotoxic cardiomyopathy, a rare but potentially fatal form of dilated cardiomyopathy (54). However, secondary to their neurocognitive and neuromotor deficits, which are associated with decreased ability to communicate and ambulate, patients with MCT8 deficiency cannot report clinical symptoms and do not present with decreased capacity for physical activity.

Atrial fibrillation is considered one of the most common cardiac complications of thyrotoxicosis and has been linked to an increased risk of thromboembolism. Increased risk of atrial fibrillation has specifically been linked to increased T3 and increasing age. Atrial fibrillation is uncommon under 40 years of age, but one study indicated that it occurs in up to 40% of individuals over the age of 60 with non-syndromic hyperthyroidism (59, 60).

Prolonged QT interval is associated with an increased risk of arrhythmias and cardiovascular mortality. Hyperthyroidism due to Graves disease has been associated with prolonged corrected QT interval, with a strong correlation between free serum T3 and QT interval. This is normalized in these patients upon return to the euthyroid state (61, 62). Conversely, other research has found the QT interval to be shortened in thyrotoxicosis and significantly negatively correlated with free T3 concentration (55). Of note, a shortened QT interval is associated with an increased risk of cardiac arrhythmia and sudden cardiac death (63, 64). This unclear relationship between thyroid function and cardiac electrophysiology may arise from the complex effect of TH on the mechanisms of rhythm generation (58). Thus, evidence does not strongly support that repolarization abnormalities mediate the increased cardiovascular risk in thyrotoxicosis. In MCT8 deficiency, prolonged QT interval has been observed in a small number of patients and could be considered a risk factor for sudden cardiac death in these individuals (19).

TH action in the heart is dependent on serum T3. Therefore, the heart will still express a hyperthyroid phenotype if TSH and T4 are normal and T3 levels are raised (52). This may be particularly important for patients with MCT8 deficiency, who characteristically show elevated T3, but with TSH within reference range. One of the major causes of mortality for individuals with MCT8 deficiency is “sudden death” (19). This may be attributable to cardiac abnormalities, as observed in individuals without MCT8 deficiency, due to chronic thyrotoxicosis, although further research is needed to delineate this relationship (9, 29, 65, 66).

Tachycardia at baseline is common in patients with MCT8 deficiency. Although patients are often hyperstimulated and restless, tachycardia is still present when patients are in a more restful state. Comprehensive data on the specific cardiovascular impacts of MCT8 deficiency are sparse; however, a range of cardiac and vascular abnormalities have been reported in these individuals (19). In a retrospective, natural history study, the most commonly reported clinical cardiovascular features were premature atrial contractions (n = 34/45 patients assessed; 76%) and elevated systolic blood pressure (n = 25/47; 53%) (19). However, the clinical response to elevated T3 in the heart appears to vary among individuals with MCT8 deficiency. For example, tachycardia was observed in 31% (n = 20/64) of patients in the natural history study, compared with 43% (n = 19/44) of patients in the TRIAC Trial I study (19, 67). In contrast, a small cohort study of 4 patients with MCT8 deficiency (age range 0.58-7.16 years) reported no cases of tachycardia (40).

A link between these symptoms and TH levels is yet to be fully established. Reduction in free serum T3 has been associated with reduced heart rate in patients with MCT8 deficiency, correlating this feature with T3 levels (67, 68).

Data on the specific mechanisms of cardiovascular thyrotoxicosis in individuals with MCT8 deficiency are limited. However, in patients with resistance to thyroid hormone β (RTHβ), elevated levels of circulating TH result in a hyperthyroid cardiac state. Clinically, individuals with RTHβ often experience tachycardia or tachyarrhythmias, with some developing atrial fibrillation and impaired cardiac function (29, 66, 69). Patients with homozygous TRβ variants may progress to life-threatening or fatal cardiomyopathy (66). While the long-term impact of chronic hyperthyroidism and tachycardia in patients with MCT8 deficiency remains unclear, individuals with RTHβ face an increased risk of all-cause mortality, major cardiovascular events, and a heightened risk of atrial fibrillation and heart failure, with the median age of the first adverse event reported at 56 years (29). Based on these findings, cardiac monitoring should be considered an integral part of care for individuals with MCT8 deficiency (19, 27, 49).

### Gastrointestinal manifestations

Patients with thyrotoxicosis often experience gastrointestinal (GI) abnormalities, and symptoms include abdominal discomfort and diarrhea (70). Up to 25% of cases of thyrotoxicosis have mild to moderate diarrhea with frequent bowel movements, some degree of fat malabsorption, intestinal hypermobility, and impaired anorectal physiology (70). However, individuals with MCT8 deficiency more frequently suffer from constipation, a gastrointestinal consequence better aligned with hypothyroidism (10, 19).

Although rare, patients with thyrotoxicosis may experience dysphagia, potentially due to neurohormonal dysregulation and myopathy involving the striated muscles of the pharynx and the upper third of the esophagus. Furthermore, the oropharyngeal phase of deglutition may be impaired, predisposing patients to nasal regurgitation and aspiration pneumonia. It is thought that correcting the underlying thyroid disorder is sufficient to reverse the dysphagia (70).

Impaired swallowing function and aspiration pneumonia are often reported in individuals with MCT8 deficiency (19). This has not been directly linked to thyrotoxicosis and could be attributed to their axial hypotonia and muscle wasting. However, impaired swallowing should be closely monitored in MCT8-deficient individuals as it may worsen other consequences of thyrotoxicosis. One of the leading causes of premature death in these individuals is pneumonia caused by recurrent infection or aspiration due to impairment in swallowing function. Impaired swallowing associated with insufficient calorie intake, compounded by their increased basal metabolic rate, could further exacerbate the low body weight of individuals with MCT8 deficiency (9). A study assessing swallowing in an individual with MCT8 deficiency used flexible endoscopic evaluation of swallowing (FEES) to reveal a global weakness in motor swallowing functions, consistent with central hypotonia and low body weight. Routine investigation with FEES of MCT8-deficient patients with low body weight, recurrent pulmonary infections, and swallowing difficulties could identify individuals who can be supported by feeding tube placement (71).

Gastroesophageal reflux disease is prevalent in individuals with MCT8 deficiency. This is often chronic and requires treatment (19). It is unclear whether this is linked to the thyrotoxic state, although it is likely exacerbated by muscle wasting, alterations in GI motility, and immobility.

### Impact on bone

TH signaling plays an important role in the normal development and maintenance of bone. Although TH is necessary to achieve peak bone mass during development, excess TH action in childhood has been linked to accelerated skeletal development and premature fusion of growth plates and cranial sutures, the latter presenting as craniosynostosis (72). High free serum T4 concentration (even within reference range) during childhood is associated with lower bone mineral density and content, although no evidence of increased fracture risk has been demonstrated (73). In adults, thyrotoxicosis is associated with accelerated bone turnover and loss of bone mineral density, resulting in increased fracture risk and osteoporosis (72).

The impact of thyrotoxicosis on bone is also evident in studies of patients with RTHβ. Adults with RTHβ exhibit lower bone mass, while biochemical abnormalities have been reported in children. Patients of all ages show calcium levels at the upper normal range, low inorganic phosphate, and elevated fibroblast growth factor 23 levels, potentially indicating increased bone turnover, resulting in bone loss and increased calcium flux into the circulation (74).

Thyrotoxicosis may result in increased fracture risk for individuals with MCT8 deficiency. Global *Mct8* knockout (KO) mice show low bone mass in the trabecular bone compartment, a high bone turnover state, and T3-dependent accelerated bone metabolism (75). Conditional *Mct8* osteoblast or osteoclast progenitor KO mice show increased trabecular bone in young mice, with a shift toward trabecular bone loss in the femur and spine in older mice compared with controls (76).

A small sample of patients with MCT8 deficiency aged 8 years or older were below the 5th percentile for bone mineral density. However, bone turnover markers in this study were generally within the low-normal range (19). Thus, frequent fractures in individuals with MCT8 deficiency may not be entirely attributable to the impact of thyrotoxicosis on bone. Individuals are largely immobile, which may be a more important contributing factor to fractures and decreased bone mineral density.

### Liver

Biochemical hepatic abnormalities are observed in 15% to 76% of patients with untreated thyrotoxicosis; however, acute liver failure is uncommon (77, 78). Suggested causes of liver dysfunction include direct hepatocyte injury, comorbid atrial fibrillation and heart failure, associated autoimmune conditions (particularly in the context of Graves disease), preexisting liver disease, chronic hepatitis B or C, dyslipidemia, and the use of medications, including antithyroid medications (78, 79). Mild elevations in liver transaminase levels (typically less than 4 times the upper limit of normal) often normalize in 77% to 83% of patients treated with thionamide antithyroid therapy, supporting the association of hyperthyroidism itself with mild liver inflammation (78).

In thyroid storm, acute liver failure may occur and is associated with high mortality (80). It has been suggested that increased serum T3 may directly cause mitochondria-mediated hepatocyte apoptosis, hepatic ischemia secondary to peripheral vasodilation, or excess TH causing right-sided heart failure, leading to increased back pressure and hepatic failure (77, 81). Younger age, congestive heart failure, and total bilirubin ≥3.0 mg/dL are also predictive factors for thyroid storm diagnosis among thyrotoxic patients (79).

Some patients with MCT8 deficiency exhibit abnormal liver function. Specifically, 20% to 46% of patients show elevated transaminase levels, while 88% have elevated sex hormone-binding globulin (SHBG) levels (19). SHBG is a marker of TH signaling in the liver, suggesting that MCT8 deficiency induces a hyperthyroid state in this organ. In a small subset of patients, this is accompanied by reduced total cholesterol, a clinical feature indicative of increased hepatic hormone signaling (4, 19).

### Recurrent infections

Alterations in TH levels impact the immune system, and patients with hyperthyroidism may display perturbed immune responses. This includes abnormal antibody production, increased migration of polymorphonuclear leukocytes, lymphocyte proliferation, and increased reactive oxygen species (ROS) production by macrophages. Furthermore, reduced pro-inflammatory markers and lower antioxidant enzyme activity have been reported (82).

Individuals with MCT8 deficiency appear prone to recurrent pulmonary infections and aspiration pneumonia, leading causes of death in these patients. Although a link between this outcome and an altered immune response due to thyrotoxicosis has not been established, monitoring for the presence of respiratory infection should be considered (19).

### Sleep

Thyrotoxicosis causes sleep disturbance in a large proportion of individuals, including difficulty in falling asleep, difficulty staying asleep, and reduced sleep efficiency. Difficulty falling asleep in individuals with thyrotoxicosis is associated with hyperkinetic features such as dyskinetic movements, hypnic clonus, myoclonus, and exaggerated startle reflex. Patients with tremor are more likely to have difficulty staying asleep (83). Sleep disturbances are also common in patients with Graves disease, and their severity has been correlated with free serum T4 (84).

Sleep disturbances in patients with MCT8 deficiency include insomnia and parasomnia, further exacerbated by high rates of dystonia and spasticity (19). Some individuals with MCT8 deficiency have repetitive sleep starts (RSS) during the onset of non-rapid eye movement (nREM) sleep. RSS are characterized by brief, clustered bilateral limb contractions recurring for several minutes, which may induce arousal and sleep fragmentation (85).

Sleep disturbance in thyrotoxicosis may add to poor physical outcomes and a reduced quality of life for the patient and caregivers. Disturbed sleep may further contribute to glucose dysmetabolism and have adverse consequences on cardiovascular performance (83).

### Management and monitoring

Managing MCT8 deficiency-induced thyrotoxicosis presents a unique therapeutic challenge: treating systemic TH excess without worsening the brain's localized TH deficiency. Traditional antithyroid medications that aim to suppress TH production can be harmful to individuals with MCT8 deficiency. They further deprive the brain of TH, potentially worsening neurological outcomes (49, 86).

Key recommendations set out by the European Thyroid Association (ETA) recognize that the main aim of MCT8 deficiency treatment is to increase TH action in the brain while alleviating thyrotoxicosis in tissues outside the brain (49). The 2024 ETA guidelines on genetic disorders of thyroid hormone transport, metabolism, and action, including MCT8 deficiency, recommended tiratricol as long-term therapy for all patients with MCT8 deficiency (49). Evidence for such treatment has been reported in case series, cohort studies, and one phase 2 clinical trial (Table 1). The use of conventional antithyroid medications, such as levothyroxine, propylthiouracil, and methimazole, is limited by a lack of consistent clinical benefit, or indeed a worsening of symptoms or significant safety concerns (27, 39, 87, 88, 91-94). T3 analogues that bypass MCT8, such as DITPA and particularly tiratricol (Emcitate®), show greater promise, with Emcitate® approved by the European Commission (EC) for the treatment of peripheral thyrotoxicosis in patients with MCT8 deficiency, from birth (49, 91, 95).

**Table 1.** Summary of evidence for the treatment of patients with MCT8 deficiency

Treatment	Evidence and outcomes
Levothyroxine (LT4)	Treatment with LT4, a synthetic T4 replacement medication, has been described over wide dose ranges in case reports. LT4 treatment has not been shown to improve neurodevelopment, and in some cases, it has exacerbated the hyperthyroid state in peripheral tissues (39, 87, 88).
Propylthiouracil (PTU) with levothyroxine (LT4)	PTU is an antithyroid drug that interferes with the conversion of T4 to T3. The addition of PTU to LT4 treatment has been shown to ameliorate some thyrotoxic symptoms in select patients, increasing body weight, reducing heart rate, and normalizing SHBG concentrations (8, 89). In contrast, in a study of 12 male patients with MCT8 deficiency, combined treatment with PTU and LT4 normalized serum T3, free T4, and total T4, with TSH levels becoming undetectable. In this study, there were no statistically significant changes in the metabolic or physical parameters studied, such as heart rate and weight (90). PTU causes potentially serious hepatic side effects: it holds a US Food and Drug Administration black box warning and is contraindicated for use in pediatric patients (91, 92). Therefore “block and replace’ therapy with combined PTU and LT4 cannot be recommended as the risk of adverse reaction to PTU outweighs the benefit. Further studies are needed to determine if “block and replace’ should be considered as a therapeutic option (93).
Methimazole (MMI) and carbimazole	MMI and carbimazole (a precursor of MMI) are antithyroid drugs that block the synthesis of THs by inhibition of the enzyme thyroid peroxidase. They are ineffective in treating MCT8 deficiency as they do not reduce serum T3 levels (they do not inhibit DIO1 activity) (27, 94).
Diiodothyropropionic acid (DITPA)	DITPA is a T3 analog that can enter the brain in an MCT8-independent manner. DITPA has been shown to normalize TH levels in patients with MCT8 deficiency. In tissues outside the brain, it decreases serum T3 levels by lowering DIO1 activity and thus preventing T4 to T3 conversion. DITPA was shown to have mixed effects on peripheral symptoms in patients with MCT8 deficiency, including a reduction of sleeping heart rate (3/4 cases) and improvement in body weight (2/4 cases) (91).
Tiratricol (triiodothyroacetic acid, TRIAC, Emcitate®)	Tiratricol is a naturally occurring T3 analog that enters the brain in an MCT8-independent manner. Tiratricol (Emcitate®) is approved by the European Commission (EC) for the treatment of peripheral thyrotoxicosis in patients with MCT8 deficiency, from birth (95). Ongoing clinical trials include data analysis for prospective phase 2 and phase 3 clinical trials that are still open for recruitment. Current data show that treatment with tiratricol had beneficial effects, including increased body weight, lower resting heart rate, reduced premature atrial contractions, reduced systolic blood pressure, and reduced SHBG (49, 67, 68).

#### Monitoring symptoms of thyrotoxicosis in patients with MCT8 deficiency

Treatment, monitoring, and symptom management require close collaboration between endocrinologists, neurologists, nutritionists, and other specialists to address the complex and multifaceted needs of patients with MCT8 deficiency. The specific symptomatology and requirements of the individual patient should be considered when developing a treatment plan, accounting for the heterogeneity of the disorder between individuals and throughout their lives. Patients with MCT8 deficiency have subclinical thyrotoxicosis, presenting with tachycardia and an increased metabolic rate associated with failure to thrive, but do not appear clinically hyperthyroid, with an absence of jitteriness, tremor, and restlessness.

Management strategies for thyrotoxicosis in MCT8 deficiency should focus on alleviating symptoms arising from thyrotoxicosis in tissues outside the brain, while minimizing the risk of complications, through supportive care strategies to improve the quality of life for individuals and caregivers.

#### Metabolism and muscle wasting

Low body weight and failure to thrive often prompt the first presentation to an HCP. Assessment of body weight is recommended every 3 months in infants and children. The ETA also recommends a nutritional assessment (49). One recent prospective registry study found that only 19% (6/32) had a pediatric gastroenterologist as part of their care team, and 31% (11/36) had received advice from a dietitian or nutrition expert (9, 10, 49).

A feeding tube (gastric tube; g-tube) may address issues related to low body weight caused by a combination of swallowing difficulties, risk of aspiration, and the increased caloric intake required to maintain weight associated with thyrotoxicosis and hypermetabolism. The need for a feeding tube should be evaluated by a gastroenterologist, with FEES providing critical insights into each individual's swallowing capabilities (71). Although feeding difficulties were a primary concern for patients in one survey, only 2 of the 16 patients assessed had a feeding tube (10).

Patients with MCT8 deficiency often present with muscle wasting and weakness, likely linked to malnutrition and elevated serum T3 levels. Some patients exhibit elevated serum creatine kinase levels, but it remains unclear whether these mild to moderate elevations are clinically significant (19).

#### Gastrointestinal issues

Monitoring dysphagia is also an important consideration for patients with MCT8 deficiency. Dysphagia can contribute to the inability to gain weight and increase the risk of aspiration pneumonia. Swallowing function should be regularly assessed by speech therapy and radiographic swallowing studies (9, 19, 71, 96). Aspiration pneumonia is a known cause of premature mortality in patients with MCT8 deficiency. Although this has not been directly linked to thyrotoxicosis, many symptoms are considered risk factors, including dysphagia, low body weight, and muscle wasting and dysfunction. Amelioration of these symptoms of thyrotoxicosis may reduce the risk of aspiration pneumonia in these patients. Gastroesophageal reflux disease and silent micro-aspirations are specific risk factors for the development of pneumonia in infants and children, particularly those with learning disability. Caregivers and HCPs should be aware of this increased risk and consider monitoring for abnormal cough, excessive secretions, and fever (96). These monitoring assessment results should inform the timing of a feeding tube referral.

#### Skeletal abnormalities

HCPs and caregivers for patients with MCT8 deficiency should be aware that these individuals are at increased risk of osteoporosis, scoliosis, hip subluxation, and fractures. Patients should have baseline radiographs of the spine and hip to establish risk. Monitoring of bone mineral density using a dual-energy x-ray absorptiometry (DEXA) scan can evaluate fracture risk (49).

#### Cardiovascular abnormalities

Sudden death is a common cause of mortality in patients with MCT8 deficiency and may be linked to underlying cardiovascular issues (9, 65). Cardiac manifestations of thyrotoxicosis are wide-ranging; patients may appear asymptomatic, yet tachycardia is present in many patients, and all patients are at increased risk for tachyarrhythmias and cardiomyopathy (19, 54). An international registry study indicated that only 6.25% (2/32) of patients with MCT8 deficiency were under the care of a cardiologist (10).

Patients with MCT8 deficiency should be monitored for tachycardia, conduction abnormalities, and systolic hypertension. Standard cardiovascular monitoring, including regular assessment of heart rate and blood pressure, is essential. The dystonic, hyper-responsive, and reflexic responses to stimuli present challenges to the clinical evaluation of heart rate and blood pressure (49).

Electrocardiographic (ECG) monitoring should be used to evaluate cardiac arrhythmia and dysfunction. In patients with MCT8 deficiency, cardiac monitoring using a 14-day ECG patch could provide useful measures of cardiac function. These patches are well tolerated, may be more sensitive for detecting cardiac arrhythmia, and could be useful for continuous monitoring of patients. ECG patches and standard electrocardiograms should both be considered key elements in the care plan of adults with MCT8 deficiency, given the increased risk of atrial fibrillation with age and the increased risk of cardiac events in individuals with a prolonged thyrotoxic state (97). Echocardiography should also be used to further assess abnormalities in cardiac structure and function (54).

#### Establishing monitoring parameters

In MCT8 deficiency, no minimal clinically important difference has been defined for any of the parameters mentioned, largely due to the rarity of the disorder and the heterogeneity of presenting symptoms. Therefore, it is essential to establish baseline measurements for individual patients and frequently monitor their condition. In patients older than 3 to 6 months of age, the initial evaluation of endocrine status should include TH levels, with increased free serum T3, low FT4, and normal to slightly elevated TSH being pathognomonic for the condition. These parameters are often established during the initial diagnostic workup before *SLC16A2* variant testing (49). Normalization of serum T3 may serve as a marker of treatment efficacy in patients with MCT8 deficiency (67, 68). However, the degree of abnormality in thyroid function testing does not always correlate with symptom severity. Additional serum markers of thyrotoxicosis can include elevated SHBG, low creatine kinase, low creatinine, low total cholesterol, and elevated alanine aminotransferase (49).

## Conclusions

The relationship between MCT8 deficiency and thyrotoxicosis is complex. More research is needed to strengthen correlations between TH status, symptoms observed in other hyperthyroid disorders, and those specific to MCT8 deficiency. Key clinical manifestations of thyrotoxicosis in patients with MCT8 deficiency include weight loss or failure to gain weight, muscle wasting, and cardiac abnormalities, and an increased risk of cardiomyopathy. Chronically elevated serum T3 affects multiple organs, yet there is limited understanding of how these effects converge to contribute to the poor outcomes seen in patients with MCT8 deficiency. Assessing the long-term effects of sustained T3 elevation throughout the lives of these patients is an important area in need of greater research, as prolonged TH elevation has been linked to adverse outcomes in other disorders of TH transport (29, 30).

Symptoms and long-term sequelae of thyrotoxicosis in tissues outside the brain are common in individuals with MCT8 deficiency and contribute to a reduced quality of life and potentially premature mortality (19). Alongside aiming to improve neurodevelopmental outcomes, alleviating symptoms of thyrotoxicosis in tissues outside the brain should be considered a primary treatment goal in patients with MCT8 deficiency, with HCPs and care providers referring patients to specialists such as cardiologists and gastroenterologists (9, 10, 49, 86).

Early diagnosis and reduced access to effective therapy remain key challenges in improving the quality of life for patients with MCT8 deficiency and their caregivers. However, reports show that the time between symptom onset and diagnosis has shortened, indicating growing awareness of this disorder. Increased recognition and the early implementation of monitoring and treatment plans are critical for improving patient outcomes (10).

## Data Availability

This article is a narrative literature review and does not involve the generation or analysis of new datasets. All data discussed are derived from previously published studies, which are cited within the manuscript.

## Abbreviations

DIO: deiodinase
ECG: electrocardiography
ETA: European Thyroid Association
FEES: flexible endoscopic evaluation of swallowing
GI: gastrointestinal
HCP: healthcare professional
MCT8: monocarboxylate transporter 8
rT3: reverse triiodothyronine
RTHβ: resistance to thyroid hormone β
SHBG: sex hormone-binding globulin
SLC16A2: solute carrier family 16 member 2
T3: triiodothyronine
T4: thyroxine
TH: thyroid hormone
TR: thyroid hormone nuclear receptor
TSH: thyrotropin (thyroid-stimulating hormone)
